# How Do Persons Who Inject Drugs Experience Care From Nurses in Hospital Settings? A Qualitative Study

**DOI:** 10.1177/23333936241240795

**Published:** 2024-04-03

**Authors:** Kjerstine Solheim, Marit Hegg Reime, Leslie S. P. Eide

**Affiliations:** 1 UIT The Arctic University of Norway, Tromsø, Norway; 2 Western Norway University of Applied Sciences, Bergen, Hordaland, Norway; 3 Lovisenberg Diaconal University College, Oslo, Norway

**Keywords:** people who inject drugs, PWID, people who use drugs, PWUD, hospital, nursing care, patient experience, Norway

## Abstract

People who inject drugs (PWID) are at increased risk of acute and chronic health outcomes and in need of in-hospital healthcare services. This study aims to give insight into how PWID experience care from nurses in hospital settings. We used a qualitative descriptive design and applied reflexive thematic analysis to 11 individual semi-structured interviews with PWID. Our analysis generated the following main themes: (1) diminishment and distance—always just a drug addict, (2) gratitude—equal care not taken for granted, and (3) vulnerability—already carrying a heavy burden. Our findings reveal a complex, nuanced narrative regarding participants’ experiences of nursing care and highlight the importance of enhancing knowledge, understanding, empathy, and communication skills when nurses encounter PWID. Our research suggests that patients’ vulnerability resulting from previous experiences defined their perception of quality of care. Insight from this study provides valuable knowledge about how to enhance nursing care for PWID.

## Introduction

The injection of illicit drugs is a global public health issue, with an estimated 15.6 million individuals engaging in this practice worldwide (Degenhardt et al., 2017, p. 86). In Norway, an estimated 8.500 residents regularly inject illicit drugs (Burdzovic, 2022), resulting in a significant burden of morbidity and mortality in this population (Gjersing, 2018). Illicit drug use poses the risk of adverse health outcomes with both acute and chronic health damage, and death (Babor et al., 2019; Crepet & Sargent, 2022; European Monitoring Centre for Drugs and Drug Addiction, 2023; Haber et al., 2009; Ozga et al., 2022). Opioids, amphetamines, and cocaine pose the highest risk of injury, especially when injected (Babor et al., 2019). As a result, people who inject drugs will need treatment in hospital settings (Haber et al., 2009).

Injection drug use is defined as taking drugs subcutaneously, intramuscularly, or intravenously (Gjerde et al., 2023). In global terms, the most common injected drugs are opiates and stimulants (Degenhardt et al., 2017; European Monitoring Centre for Drugs and Drug Addiction, 2023; Gjerde et al., 2023). In the Norwegian context, the most common injected drugs are heroin (opiate) and amphetamines (stimulants) (Gjerde et al., 2023) and several studies show that over 70% of people who inject drugs are males (Gjersing & Helle, 2021; Opheim et al., 2024). According to the most recent data from the Norwegian Institute of Public Health (2023), there were 321 overdose deaths (32% of them females) registered in the country in 2022. Opioids such as morphine, codeine and oxycodone account for a high proportion of these deaths (26%), followed by heroine (21%). Synthetic opioids such as buprenorphine, fentanyl and pethidine are responsible for 14% of overdose deaths (Norwegian Institute of Public Health, 2023).

It has been suggested that people who use illicit drugs and are in need of hospital care experience mistrust, marginalization, stigmatization, dehumanization, negative comments, and differential treatment from nurses compared to other patients (Bearnot & Mitton, 2020; Biancarelli et al., 2019; Dion, 2019; Paquette et al., 2018). This can ultimately result in undertreatment and mistreatment in hospital settings (Crepet & Sargent, 2022; van Boekel et al., 2013). Because of their experiences, people using illicit drugs may postpone or fail to seek proper treatment despite their need, and when in contact with healthcare providers, they may use strategies to avoid stigmatization (Ozga et al., 2022). Such strategies may include underreporting pain and avoiding the disclosure of drug use (Biancarelli et al., 2019; Paquette et al., 2018).

A Norwegian survey (Krokmyrdal & Andenæs, 2015) examining nurses’ competence and attitudes regarding pain management for people with opioid addiction found that 87% of the nurses reported low competence in pain management for people with opioid addiction. Many nurses believed that these individuals exaggerated their pain in order to obtain opioids and that they did not report the effects of the drugs honestly. Other studies reveal negative attitudes toward people who use illicit drugs and that healthcare personnel finding working with this group to be unmotivating and unsatisfying (van Boekel et al., 2013).

In the Nordic tradition of care, represented by the three professors in caring science Katie Eriksson (Finland), Kari Martinsen (Norway), and Karin Dahlberg (Sweden), the concept of caregiving is understood as a natural phenomenon, with the patient’s world, vulnerability, health and suffering as its core (Arman et al., 2015). Caring science and evident caring acts begins in a perception of the lived experience of the patient, and is based on interest, respect and understanding of the patient’s world (Arman et al., 2015). The Norwegian philosopher Kari Martinsen emphasizes that care always requires two participants, where one has thoughtfulness and concern for the other (K. Martinsen, 2003, p. 69). As a consequence of someone’s suffering, another will grieve by suffering with, and by relieving the suffering. Caring is a trinity as it is simultaneously relational, practical, and moral (Alvsvåg, 2017). Through a heartily participating eye the nurses put themselves into a position where they may become worthy of the trust of the other (K. Martinsen, 2006, p. 71). Martinsen described compassion as implicit in the caregiver’s ability to “see with the heart’s eye” (Arman et al., 2015).

The values of person-centered care (PCC) align with patients’ perceptions of quality care (Edvardsson et al., 2017; Pratt et al., 2021). In PCC, the patient is an active participant in their own care and in the decision-making process surrounding it (Kwame & Petrucka, 2021). PCC is based on holistic understanding, which enables the nurse to better comprehend how illness affects the whole person and thus respond to the patient’s foundational care needs (Morgan & Yoder, 2012). PCC care requires an empathetic, respectful, engaged, and relational approach, where the individual is at the center and the significance of their context, history, and family relationship is recognized (Håkansson Eklund et al., 2019).

Little is known about how persons who inject drugs (PWID) experience care from nurses in hospital settings, and no Scandinavian studies focusing on this topic have been found. Therefore, the aim of this study is to explore how this patient group experiences receiving care from nurses in hospital settings in Norway.

## Methods

### Study Design

This study was conducted within the big Q qualitative paradigm, which recognizes the inevitable subjectivity of the researcher and the active role researchers play in knowledge production (Braun & Clarke, 2022a, pp. 7–8; Gough & Madill, 2012). We chose a qualitative descriptive design, as described by Sandelowski (Sandelowski, 2000, 2010). This design was chosen as we intended to “give voice” to the participants and hereby provide data-near findings (Sandelowski, 2000), with the goal of providing rich descriptions of the participants’ experiences (Braun & Clarke, 2013).

### Recruitment, Participants, and Setting

Informants were recruited in two low-threshold healthcare centers for people who use drugs in two larger Norwegian cities between October 2022 and February 2023. Low-threshold centers for people who use drugs receive support from the government and can be found around the country. Their aim is to improve life conditions for people using illicit substances by offering health services, overdose prevention measures, advice, individual follow-up, housing, work inclusion, and follow-up for relatives. These centers also function as a social arena for the target group. All centers have food service and an appointment is not necessary (Helse- og omsorgsdepartementet, 2010).

These low-threshold healthcare centers for people who use drugs provided valuable input on the recruitment strategy, as PWID are considered a challenging group to reach (Batista et al., 2016; Faugier & Sargeant, 1997).

The first author contacted the low-threshold centers for people who use drugs and, informed about the study both verbally and in writing. Recruitment strategies and the practical conduct of the potential interviews were also discussed during these meetings.

We promoted the study with posters and employed purposeful sampling (Patton, 2002) to recruit individuals satisfying the following criteria: (1) admittance into a hospital setting within the previous 3 years, (2) regular injection of illicit drugs at the time of hospitalization, and (3) aged >18 years at the time of hospitalization. Staff from the low-threshold healthcare center for people who use drugs who helped recruiting participants were informed that diversity in gender and age was desired. Individuals agreeing to participate were then put in contact with the interviewer (first author), who provided verbal and written information about the study before the informants gave their written informed consent to participate. Five of the participants were interviewed at the first center. The interviews were conducted on site and in private offices provided by the low-threshold healthcare centers for people who use drugs. Following a recommendation by the second healthcare center and an on-site patient representant, the final six participants received modest economic compensation (a voucher for NOK 200, equivalent to less than USD 20).

The study population consisted of 11 PWID, all of them originally from Norway, with Scandinavian ethnicity, and nine of them male. Five participants reported a preference for opiates, three for amphetamines, and three for both substances in combination. The average duration of injection drug use was 19.3 years (range: 6–40 years). The participants’ ranged in age from their thirties to their sixties. Over the previous 3 years, the participants had been admitted to hospital settings between one and six times (average 2.7 admissions per person).

### Data Collection

We employed a topic-based semi-structured interview guide (Braun & Clarke, 2013, p. 84). A pilot interview was conducted with a person who had previously injected drugs and who is now a patient representant. Even though data from this pilot interview was not included in the analysis, this insider perspective was considered useful for improving the interview guide and the quality of the interviews (Brett et al., 2014).

The interview guide was used flexibly to obtain rich data on the participants’ perspectives and to allow the participants opportunities to address issues important to them (Braun & Clarke, 2013, p. 81). The first author encouraged the participants to speak freely about their experiences of contact with nurses in hospital settings. Each interview started with participants being asked about their most recent experience as an inpatient in a hospital. After their initial contact was established, we proceeded to talk about the participants’ experiences with hospital care and their interaction with nurses. The interviews lasted between 10 and 105 min (mean 45 min), and they were audio recorded and transcribed by the first author soon afterward. The transcribed material consisted of 91, single-spaced, pages in a Word document.

We continually assessed the information power (Braun & Clarke, 2022a, p. 16; Malterud et al., 2016) and deemed the data to be sufficiently rich after 11 interviews.

### Analysis

We employed reflexive thematic analysis (Braun & Clarke, 2022a, 2023) informed by a critical realist onto-epistemology (Braun & Clarke, 2022a; Maxwell, 2012, p. vii). Given the aim of exploring the participants’ contextual and situated experiences, we took an experiential orientation as we wanted to give voice to the participants and provide rich descriptions of their experiences (Braun & Clarke, 2013, p. 24). We used an inductive, bottom-up approach in the data analysis (Braun & Clarke, 2022a), approaching the data with an interpretive lens. This approach recognizes that data and facts are products of interpretation, thus we adopted a reflexive methodology to critically reflect on our own impact and influence on the analytical process (Alvesson & Sköldberg, 2017, pp. 10–11; Braun & Clarke, 2023). Reflexivity involves critical self-reflection throughout the analytic process (Braun & Clarke, 2022a).

We followed Braun and Clarke’s (Braun & Clarke, 2022b, pp. 35–36) six phases: (1) acquiring familiarity with the dataset; (2) systematic coding; (3) generating initial themes; (4) developing and reviewing themes; (5) refining, defining, and naming themes; and (6) writing-up.

We began the familiarization phase by listening to the audio-recorded interviews and reading the transcripts several times. In this phase, we practiced reflexive journaling by recording interpretations of the material, questioning assumptions, and reflecting on our interpretations (Braun & Clarke, 2022b, p. 19). In the initial coding phase, we systematically searched for data relevant to the research question. The coding process took an inductive approach as it was data-driven (Braun & Clarke, 2022b, p. 10). Thereafter, we used the codes to form three conceptual themes followed by four subthemes. In line with Braun and Clarke (2022b), the analytic process was recursive rather than linear, as we went back and forth, questioning our own assumptions and searching for new insights (Braun & Clarke, 2022b, p. 36, 2023). The codes and themes are a result of our active engagement with the collected data. Therefore, to ensure trustworthiness and quality, continual reflexivity was of the greatest importance in this part of the process (Braun & Clarke, 2022b, 2023, p. 15).

The analysis was conducted primarily by the first author, who herself is a nurse with experience working with the PWID patient group in hospital settings. Having been a registered nurse for 10 years prior to transitioning into teaching at the university level, the author undertook this study out of a personal interest in how PWID perceive their interactions with nurses in hospital settings. During the years she worked in a gastroenterology department, she developed a specific interest in patients with liver disease, such as hepatitis C. These patients often had a history of injecting drugs. The focus arose from her concern about social inequalities in healthcare. Her prior experience of the subject proved both challenging and beneficial in the analytical process, as the process of reflexivity required her to challenge her pre-existing beliefs. This was accomplished through reflexive journaling and discussions within the research team.

### Ethics

The Norwegian Agency for Shared Services in Education and Research has approved the study (reference number 702266). The Regional Committee for Medical and Health Research evaluated the study and deemed it to be outside the scope of its mandate under the Health Research Act (reference number 480806). Local data protection officials in the cities where the data collection was performed approved the protocol for privacy and recruitment strategy.

All participants received verbal and written information about the study before providing their written informed consent to participate. They were informed of their right to withdraw from the study and were given necessary contact information to do so. Ensuring the confidentiality and privacy of the participants was of the highest priority throughout the study process. Some adjustment of potentially identifying details in the participants’ stories was necessary, and we chose not to include any demographic information. Quotes are presented without numbering or pseudonyms, to prevent readers from “tracking” participants through the article. These alterations are not found to distort scientific meaning.

Audio files were deleted as soon as data transcription was completed, in accordance with the guidelines of the research institution and its data protection officer.

### Study Rigor

According to Levitt et al. (2017), the foundation of trustworthiness is methodological integrity. Methodological integrity is established when research design and procedures support the research goal, respect researchers’ approaches to inquiry, and are tailored to fundamental characteristics of the subject matter and the researchers (Levitt et al., 2017). The assessment of methodological integrity builds on two concepts: subject matter fidelity and utility in achieving goals (Levitt et al., 2017).

To ensure *subject fidelity in data collection*, we collected diverse perspectives by interviewing individuals of different ages and genders, with experience of multiple Norwegian hospitals. Additionally, we were conscious of our own influence on the data collection process. We critically evaluated the interview guide, avoided leading questions, and considered our own influence on the interview setting by employing reflexive journaling throughout. Furthermore, in pursuit of groundedness, we provided details about the analytic process and included a sample of quotes sufficient to enable readers to assess the trustworthiness of the analysis.

To enhance *utility in achieving goals in data collection*, we continually strove to collect data that would contribute to insightful analysis. We employed purposeful sampling to ensure that participants with relevant experience of the subject of interest were included. Additionally, we provided necessary information about the participants and the context of the study, enabling readers to evaluate the findings within this context.

*Utility in achieving goals in data analysis* was enhanced by asking questions capable of challenging or arguing existing representations. We achieved this by centering our focus on the concept of care and aiming to facilitate nuanced descriptions of care experiences. Moreover, we strove to present the findings coherently and show interconnectedness. We made deliberate efforts to highlight contrasts and inconsistencies within the results when presenting the themes. The aim was to enable the reader to evaluate the results in terms of coherence.

## Results

This study is based on individual interviews with 11 PWID. Our analysis generated three main themes related to the participants’ experiences of care from nurses in hospital departments: (1) diminishment and distance—always just a drug addict, (2) gratitude—equal care not taken for granted, and (3) vulnerability—already carrying a heavy burden. For the first two themes we created subthemes. As for the last theme (vulnerability), we believed subthemes would not contribute further richness to the analysis. Table 1 gives an overview of themes, subthemes, and codes.

**Table 1.** Themes, Subthemes, and Codes From the Analysis.

Theme	Subthemes	Codes (participant number)
Diminishment and distance—always just a drug addict	“You never get a second chance to make a first impression, and your first impression is your medical records”	Misunderstood (2,8) Diminished (1,2,3) Rigidity (1,2,5,9) Prejudice/judgmental (1,4,6,8,11) Presumptive/judgmental (1,4,6,8) Different rules apply (1,3,4,8)
	“No warmth, no genuineness”	Condemnation/guilt (1,4,6,8,9,11) Rejection/distance (1,4,6,9,11) Lack of knowledge and understanding (1,5,8)
Gratitude—equal care not taken for granted	“Nurses.. They are really lovely people. Mostly”	Positive perceptions of nurses in general (1,3,10) Generally satisfied (3,5,7,9,11) Unfamiliar care (8,11) Unexpected care (2,3,8,11) Serviceminided and attending to my needs (1,3,7,8) Safety and trust (1,5) Campassion/sympathy with nurses (1,6,9)
	“You notice the neighbor being treated just the same”	Unconditional care (4,11) Accept (1,2,3,4,5,8,9,10) Appreciation (1,4,8,11)
Vulnerability—already carrying a heavy burden		Expectations of guilt and blame (4,11) Understandable, in a way (3,5,11) Defenselessness (7,11) Increased sensitivity to body language/negative vibes (1,4,5,8,11) Negative expectations due to past experience in society (6,8,11) Fear of not being worthy of their care (6,8) Alone and afraid (1,6)

### Theme 1: Diminishment and Distance—Always Just a Drug Addict

This theme reflects our understanding of the participants’ experience of how their PWID status influenced nurses’ attitudes and behaviors toward them. The theme consists of two subthemes: “*You never get a* second *chance to make a* first *impression, and your* first *impression is your medical record*” and “*No warmth, no genuineness*.”

#### You Never Get a Second Chance to Make a First Impression, and Your First Impression is Your Medical Record

Most of the participants experienced that their PWID status defined them in interactions with nurses. “I’m not ME, in a way.” Many of them talked about feeling they were seen “just as a junkie.” One participant said: “It’s like that preconception they come with. You never get a second chance to make a first impression, and your first impression is your medical record.”

Participants indicated that it felt difficult to affect nurses’ perceptions of them. Their medical record somehow labeled them and shaped how they were cared for. Many described this impression as subtle and unspoken. They perceived a negative attitude, something “in the atmosphere,” that was difficult to describe. As one said:

*You feel when they have read something or know that you. . . That you are a drug addict. Because you hear it in someone. . . In something they say. Just something. So you feel sort of a negative. . . Yes. . . A negative attitude.*

Small signs perceived as a negative attitude as described here was referred to by most of the participants. They described feeling diminished and incapable of taking part in the decision-making process. As one described it, “It was like they put on blinders and said, ‘I’m a nurse, you are just a drug addict.’”

In the participants’ experience, their opinions and preferences were not taken into consideration or respected by some nurses. Some described situations concerning the placement of peripheral venous catheters, where their knowledge about the quality of specific veins was not valued. One participant described a situation where nurses were not willing to listen to her experience regarding a wound. She felt the nurses had already decided that the wound had been caused by the injection of illegal substances:

*[They] just decided that “that is how it is,” right. Like they probably did when I had the infection in my calf. Decided that I had injected there when I hadn’t. I found that so annoying, right? I can tell where I have been injecting. They should believe me when I say, “There I have injected, but there I haven’t.”*

Several participants described the rigidity of nurses’ preconceptions about them as PWID and their lack of flexibility in general. They indicated that they got the sense that different rules applied to them. These participants felt they were treated differently and received lower-quality care. One participant discussed undergoing an endoscopic procedure and how his premedication was prepared:

*(. . .) rolling up my sleeves. Then she discovers needle marks on my arms, like train tracks or whatever it’s called. But I see that it doesn’t look good. And then she said, “Waaaait a second, I’ll talk to the doctor.” Then she ran out, and then a brief time afterwards, “I’ve spoken to the doctor, and you are unfortunately not getting this injection.” So, I had a local anesthetic to drink. It was horrible.*

Several participants described the feeling that they were looked at differently and denied the treatment and care they consider standard. Some expressed regret over being honest about injecting illicit substances. Many of them also said that they could feel the change in the nurses’ attitudes once they became aware that they used and injected illicit substances. This led to frustration and hopelessness:

*Everyone will probably see me as a drug addict for the rest of my life. It’s really fucking hard to carry. It breaks my heart. It makes me. . .so sad, really.*

#### No Warmth, No Genuineness

Several participants described being seen as guilty of and being held accountable for the injuries related to injecting illicit substances. Some also described a feeling of being seen as a burden and a nuisance by the nurses. Participants described a perception that some nurses did not consider them worthy of their care, that they somehow deserved the pain they were experiencing. As one participant said:

*But I noticed quickly that it was kind of like two-sided. That they. . .yeah. . .some of them think of us as a burden, kind of. That really, we don’t have the right to life because of the choices we have made.*

Several participants described a duality in the nurses’ approach to them: either they were present and attentive, or they were not. One participant described how these often subtle differences appeared to them:

*You have those walking around like robots and (just) doing their job. . . Then you have those asking how you are and so on. That’s a huge difference.*

This shows how subtle differences in nurses’ practice of care can affect the overall experience of the care and treatment that patients receive. Several participants described these differences, and their descriptions reveal how small differences had a significant impact on their experience of care. For instance:

*It doesn’t take a lot of words. A sort of light atmosphere, instead of an annoyed atmosphere, right? Talk about nice stuff instead of just “Yes, medicine time.” Yes, and “Goodbye.” Right? There wasn’t any warmth, any genuineness there.*

Another participant states:

*I only received medical help [the second time at the hospital]. The first time I received a lot of warmth and a lot. . .in addition to medical help. And if someone wonders what works best, it works best to have both (laughter). If there was any doubt (laughter).*

These descriptions of some nurses being distanced, working without apparent compassion, and doing the bare minimum were provided by many of the participants. Some of them described the nurses’ lack of knowledge about how it is to be dependent on illicit substances and resulting in what they perceived as a lower quality of care. One participant said:

*Like I said, you have. . . Yeah traumas and diagnosis and stuff like that makes you turn to drugs, sort of. But it’s like, yeah. . . You become a product of your environment. Like me, I had a terrible childhood. That’s where it started for me.*

This participant said he felt misunderstood by the nurses and felt he was treated with distance. He related this to a lack of knowledge and therefore a misunderstanding of his situation.

### Theme 2. Gratitude—Equal Care Not Taken for Granted

Despite negative experiences with some nurses, most of the participants expressed positive perceptions of nurses in general or overall satisfaction with nurses’ care. Several described situations in which they experienced treatment with acceptance, equality, respect, and compassion by nurses. During the data analysis, we felt that these care experiences were unexpected and unfamiliar to some of the participants, and that this unfamiliarity shaped their encounters. Theme two consists of two subthemes: “*Nurses. . . They are really lovely people. Mostly*” and “*You notice the neighbor being treated just the same.*”

#### Nurses. . . They Are Really Lovely People. Mostly

In general, the participants tended to have positive perceptions of nurses. Words like “super” and “amazing” were used when describing them. One participant stated:

*And nurses, also at that small local hospital, even though I didn’t get any oxy-stuff [OxyContin]. They are really. . .really lovely people. Mostly.*

Some participants expressed concerns about nurses’ working situation, which they referred to as challenging. These participants showed compassion for the nurses and sometimes gave justifications for why nurses occasionally failed to provide quality care. One of them described a feeling of the nurses having little time to provide care and how this affected his own behavior:

*Busy, a lot like wandering back and forth, up and down. They really worked there. They did. I saw that it was very hard on them. And therefore. . .I didn’t want to bother them too much either. No.*

Several expressed gratitude for the care received and spoke about the importance of nurses’ care. One participant talked about how the quality of nurses’ care was of essential importance due to the feeling of safety and trust it gave them:

*(. . .) and chattered trivially and let you have social contact, a little input. . .so just a little bit of everything. Joking sort of. Something to laugh about, or whatever other than just today’s agenda. If you needed. . .if you had slept badly. Had nightmares, troubles breathing, been scared, had an extra tough time, then there was an extra shoulder for you. Right. The nurses do an incredibly important [job]. . ., everybody who has been in a hospital knows that. And that feeling of being taken care of. . .like “OK, here I can relax. It’s going to be fine. I’m being taken good care of.”*

This participant’s description reveals how subtle differences in the nurses’ approach make a significant difference to the overall care experience. For this participant, it made a lasting impression when nurses approached him with interest and what he described as small talk. Nurses showing interest and availability influenced his perception of the quality of care and gave an overall experience of safety during his hospitalization.

When conducting the analysis, we sensed that some participants did not expect the high level of care they received, and that this care certainly was not taken for granted. This was especially evident in an interview where the participant spoke about her typical experience of how nurses approached her:

*No, there were really just positive experiences. They were like “Hiiii, are you OK?” (gentle voice). Like. . .doesn’t she know that I’m a drug addict? They have been so positive and kind.*

It seemed that the participant expected to be stigmatized and to receive lower-quality care. During her interview, it seemed these expectations did not relate to earlier encounters with nurses but more to experiences of society in general. When she was asked about her thoughts about being admitted to the hospital in the future, she stated:

*No, I wouldn’t worry too much. Because I’m used to things going well. There are maybe one or two. . . That is. . .in a whole team, who you notice are a bit like. . .won’t really look at you and a bit like that. But otherwise almost everyone is. . . They smile and. . .yes. . .you will be treated well, so to speak.*

According to this informant, regardless of her PWID status, the nurses’ care in general is of high quality. She indicated that nurses with a negative attitude toward her because of her PWID status are a minority. This feeling seems to be shared by most of the participants as a sort of admiration for the nurses, a deep respect and appreciation, was communicated in several interviews:

*No, I just admire those people. . .who. . .have these professions. . .choose these professions. Help people like me. Trying to find the way back. . .*

#### You Notice the Neighbor Being Treated Just the Same

Most participants described experiences of acceptance from nurses. The PWID described feeling respected and the nurses being compassionate about their condition in these situations. One participant said:

*Yes, I think the nurses have been really kind to me. Because they know that I. . . They see that I’m thin. . . I’m haggard. . .many years of chaos. . .and I’m just saying straight out that I’m addicted to opiates and. . .and benzos. Have used hundreds of bottles of Xanax. So, they know.*

This participant felt that the care he received was based on acceptance and respect and an acknowledgment of his unconditional right to receive proper care and treatment. He felt that the nurses acknowledged his fundamental needs and provided the necessary care. Other participants also described being cared for in similar terms:

*I’ve been really well treated. It is. . . No, there has been so much positive and a lot of positive, nice people. Yeah. They put it all aside and. . . Why you are there and what. . . They just treat you the way they are supposed to and. . . So, you notice the neighbor being treated just the same. It is a really. . .good feeling.*

Some participants spoke about feeling appreciated by nurses and expressed their gratitude:

*Smiling, being nice. . . Encouraging. Right. If I asked about something or like. . .they went to great lengths to help me. If I was thirsty, they came running. If I didn’t want the food they served that day, they got me [something else] . . .right.*

This participant strongly expressed his gratitude for the nurses’ care and talked about how he sent flowers to the nurses’ station after being discharged. This reflects how some participants do not take high-quality care for granted. The same experience was also apparent in another interview where the participant talked about receiving highly specialized and expensive care. However, according to his perception, this was done only because of an ongoing campaign at the hospital, which had been criticized for not treating people who use illicit substances equally:

*As I’ve been told at the hospital, I was really lucky that it was sort of. . .they said that they made such a big investment because they had received criticism that drug addicts were, more or less, left to fend for themselves. So now they were really going to show that they help us too. So they went all in to help me. So I was really lucky. If it wasn’t for that campaign. . . Yeah, if not. . .then I would have had that wooden coat on (laughing).*

### Theme 3. Vulnerability—Already Carrying a Heavy Burden

Several participants discussed how experiences with stigma from society transfer to hospital settings, and to their own expectations when they are interacting with nurses. Almost all of the participants described being stigmatized in society. Some used words like “subhuman” and “second-class citizen” when reflecting on how they are perceived by society. One participant reflected on how others see him and his perception of being regarded as not equally valuable:

*That we in some way. . . Yeah, those [that] we. . .doesn’t quite categorize as “us,” I think we tend to push [them] away in some way. It’s like. . .you see a reindeer, or really all herd animals. If some of the animals behave strangely, or not like the others. . .then they will be ostracized. That’s how it is with us, too. That, sort of. . .yeah, that people will. . . [crying silently] overdoses, for instance. . . If you overdose on the street and people comes by. . .people just shy away. . . If you ask them to call an ambulance, “Go away” sort of, they hurry right past. So, it’s like, you somehow, I don’t know. . .become a subhuman.*

Some participants talked about how stigmatization and devaluation in society transfer to hospital settings and to nurses. They can easily detect the familiar experience of stigma when interacting with nurses. As one said:

*But then you notice there’s something negative there, in the background. In the back of your mind. You notice it’s there. I don’t know, but it probably relates to all those times. . . It happens all the time. Or that it just happens a lot. So just. . .also when outside as normal. You hear mothers saying “Don’t go over there. Don’t go near them.” And it sort of becomes the same way in hospitals too. A bit of chattering and gossiping.*

During the data analysis it was our impression that the participants had become more sensitive and attentive to nurses’ body language and tone as a result of these negative experiences. They discussed how they could always feel if nurses were genuine in their communication and interaction. The participants appear to have developed an increased sensitivity and attunement to signs of negative regard from nurses. Some of them reflected on how, as a result of these strong expectations, it can become a “self-fulfilling prophecy,” as expressed by this participant:

*You notice people’s attitude when you’re in hospitals. . . At least I do. . .pick up on if there is any tension in the air. But that is. . .you get used to it, because you are used to people being prejudiced and not having the best thoughts about you. Of course, at times it can be sort of self-inflicted – that you kind of expect it so much that it becomes some kind of self-fulfilling prophecy.*

When reading the interviews, we were struck by how the participants’ vulnerability could affect their perceptions and behavior in interactions with nurses. Some said they sometimes felt defenseless and small when in contact with authority figures like nurses and how that affected them.

*[you] are admitted to the hospital but feel that you are not being taken seriously because you are like . . . a second-class citizen, right? you are left with the sour aftertaste in your mouth that this is not fair, it is not right.*

Several participants said they had expected shaming and blaming from nurses. They feared they would be treated as unworthy of the nurses’ care, and some struggled with the feeling that they were just a nuisance. One described how PWID status puts one in a vulnerable position:

*Especially when something is wrong with you so. . .really. . .yeah. You are injured, ill, whatever, right? So, you are in a really vulnerable position. Wasn’t it the Vikings that pushed the sick ones off the mountains, because they didn’t have any use for them anymore? And it is sort of how you feel. That yeah. . .so it is kind of like. . .if you are treated in a proper way. . .it has everything to say. Right. You are emotionally down already, right?*

## Discussion

The aim of the study was to gain insight into PWID experiences of receiving care from nurses in hospital departments. The findings from this study tell a complex and multi-faceted story.

Several participants felt labeled and defined by their PWID status when in contact with some nurses. In their experience, this status led to their receiving lower-quality care. The negative experiences seem to be typified by nurses distancing themselves and providing a minimum level of care. These findings are consistent with international research detailing experiences of stigmatization, marginalization, dehumanization, prejudice, and discrimination in this patient group (Biancarelli et al., 2019; Dion, 2019; French et al., 2023; Lloyd, 2013; Muncan et al., 2020; Pauly et al., 2015). As suggested by our findings, negative experiences were based mainly on participants’ interaction and communication with nurses. Our findings are in line with previous research indicating that perceived level of compassion and understanding are central components in patients’ perception of quality of care (Feo et al., 2018). However, it is important to emphasize that these negative experiences are based on interactions with a minority of nurses.

Our analysis also presents positive experiences of nurses’ care and positive feelings about nurses. Our analytic story reveals gratitude and admiration for this healthcare group, and several participants described situations where they felt respected and accepted. These experiences contributed to increased feelings of safety and trust. Our findings support earlier studies showing that patients’ perception of quality of care seems to be linked to feelings of equality and to nurses seeing the person behind the drug use, to nurses seeing the patient as a whole, not as just a “problem” (Dion, 2019; French et al., 2023; Monks et al., 2013). This can be linked to the Nordic philosopher Kari Martinsen’s concept “seeing with the hearts eye,” about what “seeing” and “compassion” means in health care. According to Martinsen, practical action represents an important dimension in care approach, together with a relational and moral dimension. Opposite, through a “recording eye” nurses may see the person as an object, which may threaten the person’s integrity (E. H. Martinsen, 2011).

The themes *diminishment and distance—always just a drug addict* and *gratitude—equal care not taken for granted* stand in contrast to one another and display the differences between what participants perceive as high-quality care and low-quality care. These differences seem to be based on whether the nurse acts with compassion, attentiveness, and dedication when interacting with PWID. Through small gestures, nurses can assure the patient that the care provided was of high quality (K. Martinsen, 2006; E. H. Martinsen, 2011). These actions result in patients feeling safe and taken care of. Our findings are consistent with international research showing how, in general, patients perceive quality in nursing care (Graham et al., 2019; Wagner & Bear, 2009).

Person-centered care (PCC) is fundamental to patients’ perception of the quality of nursing care (Edvardsson et al., 2017; Kwame & Petrucka, 2021; Pratt et al., 2021). PCC emphasizes the centrality of the person within their context, with their history, individualities, strengths, and weaknesses. PCC therefore calls for a holistic approach that recognizes the patient as a whole person in his or her biological, psychological, and social context (Håkansson Eklund et al., 2019). The experiences shared by our participants and the findings of this study strongly support the principles of PCC. The theme “*Vulnerability—already carrying a heavy burden*” provides insight into how the participants’ histories and previous experiences can shape and influence their experience of being cared for by nurses. Knowledge of the effects of living with extensive stigmatization in everyday life seems important in providing high-quality care for PWID. The level of stigma experienced by PWID is extreme and greater than that experienced by other stigmatized groups (Lloyd, 2013). Injection drug use causes strong emotional reactions and people who use illicit substances are often blamed for their situation (Lloyd, 2013). It is therefore important to understand how this affects communication and interaction between stigmatized and non-stigmatized people (Lloyd, 2013). Other studies have also shown that PWID frequently pay more attention and are more sensitive to body language, tone of voice, and facial expressions because of their negative expectations, low self-esteem, and low confidence due to stigma experienced in their past (Link & Phelan, 2001; Lloyd, 2013; Muncan et al., 2020; Pauly et al., 2015).

Some of our participants fear that they are unworthy of nurses’ care. They seem concerned that, given the “self-inflicted” nature of the patients’ situation, the nurses will consider them a nuisance and therefore provide lower-quality care. Other studies have shown that PWID seem to have increased sensitivity to details in nurses’ care and are attentive in order to determine whether they are provided with equal, standard care (Dion, 2019; French et al., 2023; Lloyd, 2013; Monks et al., 2013; Muncan et al., 2020). Simple expressions of kindness and equal worth may be particularly important and comforting for PWID (French et al., 2023). Nurses need to be aware of how people with extensive experience of stigmatization, such as PWID, may be extra attentive to small nuances in order to evaluate whether the nurse is distancing herself, providing a little “extra,” or just being “normal” (French et al., 2023; Pauly et al., 2015).

### Implications for Practice

This study contributes new knowledge about the PWID experience of receiving care from nurses in hospital settings, particularly within a Norwegian context. It also provides nuanced descriptions of positive and negative care experiences, which can be valuable to nurses interacting with this patient group. The findings suggest that vulnerability and life context may influence PWID perception of nursing care quality. This offers nurses in the field valuable insight that may contribute to high-quality care for PWID. It seems important that nurses provide care based on the individual prerequisites of each patient. Individualization of care cannot be achieved without understanding the person’s background and life situation (Morgan & Yoder, 2012) or without developing a care plan in alignment with the holistic values of PCC (Håkansson Eklund et al., 2019).

Our data shows that several participants felt that nurses had limited knowledge and understanding of how their history and life-experiences had shaped them and therefore provided low-quality care. This lack of understanding might be linked to the concepts of trauma informed care (Felitti et al., 1998) and structural vulnerability (Holmes, 2011). Individuals with significant trauma histories are disproportionately represented in child welfare, criminal and juvenile justice, mental health and addictions, and social services systems (Bargeman et al., 2022; Felitti et al., 1998). Exposure to abuse, neglect, discrimination, violence, and other adverse experiences increase a person’s lifelong potential for serious health problems and engaging in health-risk behaviors, as documented by the landmark Adverse Childhood Experiences (ACE) study (Felitti et al., 1998; Goddard, 2021). One of our informants also linked his drug use to trauma in his childhood. Trauma informed care acknowledges the need to understand a patient’s life experiences in order to deliver effective care (Felitti et al., 1998), and has the potential to improve patient engagement, treatment adherence, health outcomes, and provider and staff wellness (Goddard, 2021). Establishing and maintaining communication that is open, consistent, respectful, and compassionate is vital in a trauma informed approach (Menschner & Maul, 2016). Nurses should screen persons for their trauma history and engage them in their care. Because the field of trauma informed care is relative new, building foundational awareness of trauma informed approaches should begin early in a healthcare education and be reinforced through continuing education (Menschner & Maul, 2016).

The concept of structural vulnerability (Holmes, 2011) refers to individuals or populations (for instance PWID) at risk for negative health outcomes (like infections and/or overdoses) because of their position in multiple and overlapping hierarchies that are based on political, socioeconomic and cultural definitions (Bourgois et al., 2017). When in hospital settings, PWID are in a vulnerable position (with limited economic resources, sometimes homeless and unemployed) that might also limit their commitment to healthy lifestyle modifications following discharge. Structural vulnerability proposes to shift the blame away from the victims of suffering and toward social structures leading to and perpetuating their suffering (Holmes, 2011). A way to address this lack of understanding could be by instructing current and future healthcare providers, such as nurses, about the concept of structural vulnerability. Encouraging healthcare providers’ ability to self-reflection and empathy for suffering creates structural competency and might promote encounters between patients and healthcare providers that will ultimately improve health outcomes (Bourgois et al., 2017; Gehring et al., 2022). Closer collaboration between hospital settings and low-threshold healthcare centers for people who use drugs might also contribute to better understanding of this population group.

Our findings suggest that PWID perceptions of quality in nursing care are heavily based on how nurses communicate. The nurses’ attentiveness and interest in the patient’s story and experiences are important. Our findings accord with earlier research suggesting that nurses’ attitudes and communication style can define the overall care experience for PWID (Feo et al., 2018; Graham et al., 2019). This is also in line with the ICN Code of Ethics for Nurses (2021), which states that nurses should demonstrate values of the profession such as respect, justice, empathy, responsiveness, caring, compassion, trustworthiness, and integrity.

### Study Limitations

Certain limitations must be mentioned. First, the study had relatively few participants and a minority of them were female. Even though our data has some demographic diversity, a different sample selection (e.g., in terms of gender, age, or ethnicity) might have provided additional insights. Yet, our informant group seems to give an accurate picture of sex distribution among PWID in Norway as presented in an article from Gjersing and Helle (2021). They recruited over 350 PWID from several low-threshold facilities in several Norwegian cities in Norway and found that males were disproportionally represented (76.2%) compared to females (Gjersing & Helle, 2021).

The fact that the first author conducted the interviews and had the main responsibility in performing the analysis can be considered as a limitation of this study. However, thematic analysis as presented by Braun and Clarke in 2006 and 2023 is a dynamic and reflective process and the other members of the research team contributed to the analysis by reading the interview material several times to immerse themselves into the content of the data and to become familiar with the depth and breadth of the content, in order to guide in the identification of patterns of meaning as recommended by Braun and Clarke (2006, 2022b, 2023). The senior researchers provided support and guidance as the junior researcher discusses the findings.

We recognize the complex relationship between context, language, and power in knowledge production and how these affect the meeting between the researcher and the informant (Maeland & Jacobsen, 2011). The length of the interviews varied between informants (mean 45 min), with some of them being as short as 10 min. When performing the interviews, special consideration was given to signs of exhaustion and whenever informants expressed fatigue data collection stopped. The perspective of these informants is still considered valuable and even though we were not able to ask further questions or to conduct further exploration in some areas of interest, their contributions are included in the analysis.

We are aware of the challenges associated with a person belonging to a non-stigmatized group researching the experiences of people in a stigmatized group. This may have been especially challenging in the interview setting and may have influenced what the participants chose to share. The interviewer, being a nurse herself, may also have had an impact. Interviews conducted by “inside” researchers, or non-nurse researchers, might provide additional insights. However, the interviewer’s knowledge about the study context of hospital departments and the nursing field was deemed to contribute positively, especially during the interviews in which relevant follow-up questions were asked.

## Conclusion

This study has sought to explore how PWID experience care from nurses in hospital settings. Our findings reveal a complex, nuanced story of stigmatization, diminishment, blame, and guilt, but also gratitude, equality, and acceptance. These findings highlight the importance of enhancing nurses’ level of knowledge, understanding, empathy, and communication skills when meeting PWID. Our research suggests that the patients’ vulnerability, due to their previous experiences, defined how they perceived the quality of care they received. Further research is necessary to gain a deeper understanding of the potential connection between PWID vulnerability and perceived quality of care. Studies conducted from a nurse’s perspective in a Scandinavian context may also provide valuable insight into how to enhance nursing care for this patient group.
